# Low‐Intensity Vibration Protects the Weight‐Bearing Skeleton and Suppresses Fracture Incidence in Boys With Duchenne Muscular Dystrophy: A Prospective, Randomized, Double‐Blind, Placebo‐Controlled Clinical Trial

**DOI:** 10.1002/jbm4.10685

**Published:** 2022-10-18

**Authors:** Maria Luisa Bianchi, Silvia Vai, Giovanni Baranello, Francesca Broggi, Stefan Judex, Thomas Hangartner, Clinton Rubin

**Affiliations:** ^1^ Experimental Laboratory for Children's Bone Metabolism Research Bone Metabolism Unit, Istituto Auxologico Italiano IRCCS Milan Italy; ^2^ UO Neurologia dello Sviluppo Fondazione IRCCS Istituto Neurologico Carlo Besta Milan Italy; ^3^ The Dubowitz Neuromuscular Centre UCL NIHR GOSH Biomedical Research Centre, UCL Great Ormond Street Institute of Child Health London UK; ^4^ Department of Biomedical Engineering Stony Brook University Stony Brook NY USA; ^5^ Department of Biomedical, Industrial & Human Factors Engineering BioMedical Imaging Laboratory, Wright State University Dayton OH USA

**Keywords:** BIOMECHANICS, BONE MINERAL CONTENT, BONE MINERAL DENSITY, EXERCISE, OSTEOPOROSIS

## Abstract

The ability of low‐intensity vibration (LIV) to combat skeletal decline in Duchenne Muscular Dystrophy (DMD) was evaluated in a randomized controlled trial. Twenty DMD boys were enrolled, all ambulant and treated with glucocorticoids (mean age 7.6, height‐adjusted *Z*‐scores [HAZ] of hip bone mineral density [BMD] −2.3). Ten DMD boys were assigned to stand for 10 min/d on an active LIV platform (0.4 g at 30 Hz), while 10 stood on a placebo device. Baseline and 14‐month bone mineral content (BMC) and BMD of spine, hip, and total body were measured with DXA, and trabecular bone density (TBD) of tibia with quantitative computed tomography (QCT). All children tolerated the LIV intervention well, with daily compliance averaging 78%. At 14 months, TBD in the proximal and distal tibia remained unchanged in placebo subjects (−1.0% and −0.2%), while rising 3.5% and 4.6% in LIV subjects. HAZ for hip BMD and BMC in the placebo group declined 22% and 13%, respectively, contrasting with no change from baseline (0.9% and 1.4%) in the LIV group. Fat mass in the leg increased 32% in the placebo group, contrasting with 21% in LIV subjects. Across the 14‐month study, there were four incident fractures in three placebo patients (30%), with no new fractures identified in LIV subjects. Despite these encouraging results, a major limitation of the study is—despite randomized enrollment—that there was a significant difference in age between the two cohorts, with the LIV group being 2.8y older, and thus at greater severity of disease. In sum, these data suggest that noninvasive LIV can help protect the skeleton of DMD children against the disease progression, the consequences of diminished load bearing, and the complications of chronic steroid use. © 2022 The Authors. *JBMR Plus* published by Wiley Periodicals LLC on behalf of American Society for Bone and Mineral Research.

## Graphical abstract

A double‐blind, placebo controlled trial was performed to determine if low intensity vibration (LIV) could protect the skeletal system in boys with Duchenne Muscular Dystrophy. At 14 months, trabecular bone density (TBD) in the proximal and distal tibia, as measured by QCT, remained unchanged in Placebo, while rising 4.1% and 4.5% in LIV. Height‐adjusted Z‐scores for hip BMD and BMC in Placebo declined 22% and 13%, respectively, contrasting with no change from baseline (0.9% and 1.4%) in LIV. These data suggest that non‐invasive LIV can help protect the skeleton of DMD children against the disease progression.

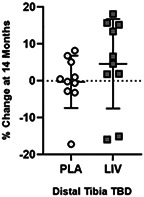

## Introduction

Duchenne muscular dystrophy (DMD) is an X‐linked recessive genetic disease due to a mutation in the dystrophin gene. DMD is characterized by severely reduced or absent dystrophin in skeletal muscles, with progressive muscle degeneration and fibro‐fatty replacement.^(^ ^1^ ^)^ Affected individuals experience progressive muscle weakness beginning in early childhood, with loss of ambulation by the age of 12 years^(^ ^2^ ^)^ and reduced life expectancy due to the progressive cardiorespiratory impairment.^(^ ^3^ ^)^ Although there is no cure, the introduction of glucocorticosteroids (GCs) in the 1990s have been shown to partially preserve muscle strength, protect pulmonary function, delay onset of ambulatory decline, reduce severity of scoliosis, and extend life.^(^ ^4^ ^)^ GC treatment of DMD is now initiated at a much earlier age, as built on clear evidence that the sooner treatment is initiated the greater its effectiveness in slowing progression of the disease,^(^ ^5^, ^6^ ^)^ and with ventilator support life expectancy can approach 40 years.^(^ ^7^ ^)^

In concert with GC treatments, novel dystrophin restoration therapies have slowed muscle collapse, and have mitigated—to a limited degree—some functional decline of DMD patients,^(^ ^8^ ^)^ yet concomitant skeletal complications persist.^(^ ^9^ ^)^ Indeed, compromised bone mineral density (BMD) and bone mineral content (BMC), are considered primary causal factors in increased occurrence of fragility fractures.^(^ ^10^, ^11^ ^)^ As compared to healthy age‐matched boys, DMD patients show a significant disparity in bone quantity and quality in the lower limbs, which in part is a consequence of reduced weight‐bearing and muscular activity on bone^(^ ^12^ ^)^ associated with this crucial period of growth.^(^ ^13^ ^)^

During the period that DMD boys retain their ability to walk, lumbar spine BMD is only slightly decreased but then drops precipitously when ambulation is lost.^(^ ^14^ ^)^ In some contrast, lower limbs are more severely affected, reflected by reduced BMD and BMC at the hip, even while walking ability is only slightly impaired. DMD boys have 30% less trabecular bone density in the tibia than healthy controls, which falls below 50% on loss of ambulation.^(^ ^15^ ^)^ This decline in bone quality is accompanied by an increase in fracture risk: Individuals with muscular dystrophies are at a 1.4‐fold increased risk of fracture when compared with population‐based controls, a risk that rises with age and triples when glucocorticoids have been used for at least 6 months.^(^ ^16^ ^)^ These fractures have a devastating impact: four of nine ambulatory DMD boys never recover to walking status after a fracture.^(^ ^14^ ^)^ Any strategy that protects bone will help reduce fractures and reduce the risk of a lifetime loss of ambulation.

It is well known that physical activity is critical in achieving and maintaining bone mass accrual across the years of growth in children.^(^ ^13^ ^)^ To a degree bone mass can be correlated to muscular strength during adolescence,^(^ ^17^, ^18^ ^)^ whereas animal studies show that lean muscle mass persists as a significant determinant of bone quantity and quality even when challenged by a dystrophic phenotype.^(^ ^19^ ^)^ Although the decline in bone status in DMD parallels deterioration of muscle phenotype, it does not appear to be directly caused by the disease itself but instead as a secondary consequence of the reduced loading that parallels muscle weakness.^(^ ^1^, ^20^ ^)^

Exercise regimens as a strategy to preserve muscle function and bone accrual in DMD children shows some promise; however, it is still unclear if this can protect from skeletal decline or if instead can lead to an increased risk of fractures.^(^ ^21^ ^)^ As a surrogate for exercise, low‐magnitude mechanical signals, delivered using low‐intensity vibration (LIV), have been demonstrated to have an anabolic effect on bone in animal models,^(^ ^22^ ^)^ driven by some degree by biasing bone marrow mesenchymal stem cells away from adipogenesis and towards osteoblastogenesis.^(^ ^23^ ^)^ High‐frequency, low‐magnitude mechanical signals are omnipresent in the functional load regime,^(^ ^24^ ^)^ arising from the dynamics of muscle contraction, and invariably decline as activity deteriorates.^(^ ^25^ ^)^ It is proposed here that introducing these low magnitude mechanical signals as a surrogate for exercise could help protect the skeleton of DMD patients, who, because of muscle decline, have reduced muscle‐induced loading of bone. The present study is aimed at evaluating the safety, tolerability, and effects on bone status of LIV in children with DMD.

## Patients and Methods

### Study design

The study was designed as a prospective, double‐blind, randomized, placebo‐controlled 12‐month trial on 20 boys with DMD. The study was reviewed and approved by the Ethical Committees of both institutions involved in subject recruitment (Istituto Auxologico Italiano IRCCS and Istituto Neurologico Besta IRCCS) and conducted according to the Clinical Good Practice rules. Recruitment for the trial was initiated before the Food and Drug Administration Amendments Act (FDAAA 801) requirement for registration with clinicaltrials.gov, so retroactive registration was made at trial completion (identifier: NCT05281120). As all potential subjects were minors, informed consent and assent was obtained from the boys' parents.

Subjects meeting the entry criteria at the screening visit were randomly allocated 1:1 to either an active LIV or placebo device designed for home use. Random numbers were generated using a random number generator by the IRCCS clinical staff, who maintained a database with even numbers being assigned to treatment and odd numbers to placebo. Subjects, parents, and study team were blinded to the active/placebo status of the device. Each enrolled subject received a LIV platform to take home. Subjects and their parents were instructed to use the platform for 10 minutes each day, scheduled at any time during the day convenient for its use. Instructions included to stand upright, in a relaxed stance, wearing only socks to cover the feet. The patients were provided a diary to record the day, time, and minutes of their platform use, and they were instructed to record any day where the LIV treatment was not used, a strategy that had shown close similarity to electronic recording of compliance in prior clinical trials.^(^ ^26^, ^27^ ^)^ Patients in both the LIV and placebo groups received weekly phone calls from the study's clinicians and hospital staff to reinforce their commitment to participating.

### Patients

Recruitment for the study was initiated through DMD family groups with clinical histories at the two enrolling institutions. Twenty ambulant boys diagnosed with DMD, aged 4–15 years (mean ± standard deviation [SD], age 7.6 ± 3.9 years), were enrolled. Baseline data are reported in Table 1. Inclusion criteria included: diagnosis of DMD; ability to stand up and walk (some balance assistance allowed, but full weight‐bearing necessary); treatment with a fixed dose of prednisone (1.25 mg/kg every 2 days, according to the treatment protocol of the Istituto Neurologico Besta IRCCS); treatment with 25‐D (calcifediol, 0.7 mcg/kg/d); and dietary calcium intake equal to the internationally recommended daily allowance (RDA). All inclusion criteria had to be met for at least 6 months before starting the study. DMD diagnosis was made at the Muscle Pathology and Immunology Unit of the Istituto Neurologico Besta IRCCS. The diagnostic criteria were based on clinical data, molecular analysis, morphological evaluation, and/or immunochemical analysis confirming the absence of dystrophin in muscle fibers.

**Table 1:** Baseline Characteristics of Body Habitus and Bone Density

Characteristic	Placebo	LIV	*p*
Age (years)	6.6 ± 1.6	9.4 ± 3.1	<0.05
Weight (kg)	20.8 ± 4.6	31.5 ± 10.2	0.06
Height (cm)	114.0 ± 12.5	127.4 ± 15.7	<0.05
Height (*Z*‐score)	1.1 ± 1.0	1.3 ± 1.4	0.45
Hip BMD (g/cm^2^)	0.530 ± 0.071	0.503 ± 0.073	0.52
Hip BMD HAZ	−1.39 ± 1.25	−2.59 ± 0.62	0.06
Hip BMC (g)	6.90 ± 2.09	8.69 ± 4.26	0.41
Hip BMC HAZ	−2.08 ± 1.28	−3.12 ± 1.12	0.21
TBLH BMD (g/cm^2^)	0.529 ± 0.053	0.596 ± 0.080	0.08
TBLH BMD HAZ	−0.33 ± 1.05	−1.24 + 0.96	0.10
TBLH BMC (g)	422.5 ± 98.7	544.5 ± 198.7	0.16
TBLH BMC HAZ	−1.07 + 0.63	−2.03 ± 0.67	<0.05
Proximal tibia trabecular bone density (mg/cm^3^)	240.2 ± 61.2	184.9 ± 46.3	0.10
Distal tibia trabecular bone density (mg/cm^3^)	203.8 ± 33.1	173.5 ± 16.1	<0.05
Tibia cortical bone density (mg/cm^3^)	1227.1 ± 40.4	1245.3 ± 58.1	0.44

### LIV platform

The active LIV platform delivered a 0.4*g* (where 1*g* is Earth's gravitational field or 9.8 m/s^2^) 30‐Hz (cycles per second) sinusoidal vibration (*n* = 10). To mask the status of the placebo platform (*n* = 10), it emitted a 500‐Hz hum through an onboard loudspeaker but produced no translational vibration signal through the plantar surface of the standing child. Peak to peak accelerations of 0.4*g* at 30 Hz require displacements of <120 μm, or the thickness of two human hairs. One hundred percent (100%) compliance would be the use of the platforms for 10 minutes each day, 7 days per week, across the length of the study. To avoid overuse, the device was restricted to a maximum of 10 minutes of LIV within any calendar day. A LIV platform designed for adults weighing between 40 and 115 kg was modified for use by children by reducing spring constants of the springs that suspend the top platen,^(^ ^28^ ^)^ allowing a weight range of 15–65 kg.^(^ ^29^ ^)^

The design of the LIV platform uses closed‐loop acceleration feedback to drive an electromagnetic actuator, ensuring a high‐fidelity sinusoidal signal,^(^ ^30^ ^)^ a design which can safely deliver these barely perceptible mechanical signals to standing subjects.^(^ ^31^ ^)^ Signals at this frequency and intensity are considered a nonsignificant risk by the U.S. Food and Drug Administration (FDA),^(^ ^32^ ^)^ and as defined by the International Standards Organization Advisory ISO‐2631, exposure to vibration signals at this frequency and magnitude is considered safe for up to 4 hours of exposure each day.^(^ ^33^ ^)^

### Evaluation program

At baseline and end‐of‐protocol, all enrolled DMD boys underwent the following evaluations: weight, height, fracture history, and an interview with a dietician to evaluate the daily intake of calcium. Weight was measured with an electric scale to the nearest 0.1 kg. All patients were able to stand up (with aid in some cases), and the standing height was measured with a stadiometer.

Baseline and end‐of‐protocol DXA and QCT bone imaging studies were performed at Istituto Auxologico Italiano IRCCS (Milan, Italy). At baseline only, bone age was evaluated with hand radiography. All the enrolled subjects underwent neurological functional evaluation at baseline and end‐of‐protocol at the Istituto Neurologico Besta IRCCS, as part of their standard clinical monitoring.

### Dietary calcium intake

Calcium intake was evaluated at baseline, administered by the same skilled dietician. Each subject had a 20‐minute to 30‐minute dietary interview, including a food frequency questionnaire and 24‐hour dietary recall, administered in a private room in the presence of one or both parents. In addition, the nutritional composition of the lunch menus (5 days/week) from the children's schools were evaluated. The frequency consumption (daily, weekly, and monthly) of each food item was evaluated. For each item, the children indicated the size of their usual meals using photographs of small, medium, and large portions. The food frequency questionnaire included 16 main food groups (eg, milk and dairy products; pasta and rice; drinks; cereals and oven products such as bread and biscuits, etc.), classified according to nutrient composition and customary use by Italian children.^(^ ^34^ ^)^ The calcium content of water (tap and bottled mineral water) was considered, obtaining the calcium content of local tap water at the patients' locations, or from the labels of the mineral waters. The analysis was performed using an Italian National Institute of Nutrition software (Winfood 1.0b), providing detailed food composition data.^(^ ^35^ ^)^

### Vitamin D and bone formation markers

From all subjects, at both baseline and 14 months, blood samples were collected between 8:00 a.m. to 9:00 a.m., after an overnight fasting. Biochemical measurements of serum 25‐hydroxy vitamin D (25‐D), 1,25‐dihydroxy vitamin D (1,25‐D), parathyroid hormone (PTH), bone‐specific alkaline phosphatase (BSAP), and osteocalcin (OC) were performed. 25‐D was quantified by radioimmunological assay (RIA; DiaSorin Inc, Stillwater, MN, USA); intraassay and interassay coefficient of variation (CV) 3.5% and 7.5%; 1,25‐D by radio receptor assay (Nichols Institute Diagnostics, San Juan Capistrano, CA, USA) intraassay and interassay CV 5.6% and 7.9%, PTH by immunoradiometric assay (IRMA; DiaSorin) intraassay and interassay CV 2.8% and 4.7%; and OC by RIA (Technogenetics, Milano, Italy); intraassay and interassay CV 3.6% and 6.9%.

### Computed tomography

Quantitative computed tomography (QCT) scans of the tibia were performed with a GE QCT (34 slices) at both legs with the same protocol in all boys. One of the coauthors (TH) analyzed these data, blinded, with special software.^(^ ^36^ ^)^

### BMD

BMC and BMD, as well as fat and lean mass, were measured by dual‐energy X‐ray absorptiometry (DXA; Hologic Discovery Horizon A densitometer) at lumbar spine, proximal femur, and total body. At Istituto Auxologico Italiano IRCCS, a strict DXA quality control procedure, including the instrument's daily phantom calibration, is standard and was regularly followed during the study. The DXA CV, with repositioning, was 0.62%–1% for spine and 0.64%–1.09% for total body, depending on age. Total body BMC, BMD, fat and lean mass were calculated excluding head (TBLH), the most appropriate measurement in a growing skeleton,^(^ ^37^ ^)^ considering the different patterns of cranial development.^(^ ^38^ ^)^ Height‐adjusted *Z*‐scores (HAZ) for BMD and BMC for spine, hip, and TBLH were calculated based on healthy boys of the same age.^(^ ^39^ ^)^

Two post hoc analyses were performed. First, changes in BMD and BMC in the spine and hip were normalized to BMD/BMC of TBLH, and second, normalized to BMD/BMC of the arm, as automatically segmented from the total body DXA measures. As the LIV platform challenges only the weight‐bearing bones and is not considered a “systemic” stimulus to the skeleton,^(^ ^32^ ^)^ BMD and BMC of TBLH and the segmented changes in the arm were considered an intrasubject measure that could be used to approximate DMD‐driven changes in the status of the skeleton that occurred across the 14 months of study, with less “exposure” to the LIV signal. As normalization of hip or spine to TBLH would include the very regions that were being examined (ie, the hip being included in TBLH would mask changes in the hip), normalization to the arm would exclude those regions being evaluated (ie, the hip would be assessed relative to a change in the arm, independent of the hip).

### Fractures

Fracture history, including circumstances, skeletal site, date, and type of intervention, was taken at baseline and updated at each clinical visit. All fractures were documented by radiography. At baseline and end‐of‐protocol, lateral radiographs of thoracic and lumbar spine were taken to evaluate the presence of vertebral fractures.

### Statistical analyses

Visual inspection of data, together with the Shapiro–Wilk test for data normality, justified the use of parametric statistics. Thus, data were expressed as the mean ± SD. For group comparisons, Student's *t* test for unpaired (placebo versus LIV) and paired (baseline versus 14 months) samples were used, as appropriate. For intragroup comparisons, changes at 14 months were expressed as absolute and relative differences from baseline values. Chi‐square tests were used to determine whether occurrence of fractures was significantly different between placebo and LIV groups. Statistical significance was defined at *p* < 0.05. Because of the small sample size, *p* values between 0.05 and 0.20 were reported as potential differences (trends).^(^ ^40^ ^)^ Values of *p* > 0.2 represented a lack of statistically significant differences. We used SPSS Statistics v. 28 (IBM Corp., Armonk, NY, USA).

## Results

Due to an illness of the lead clinical investigator (MLB) at the 1‐year mark of the first subject, the intended 12‐month protocol was extended to 14 months (~400 days), a delay incorporated into the follow‐up schedules of all boys. All enrolled patients completed the 14‐month study, and all subjects tolerated the treatment well and declared they had been happy to use the LIV platforms. Neither subjects nor parents reported any discomfort, inconvenience, or adverse effects.

### Baseline characteristics

**Fig. 1:**
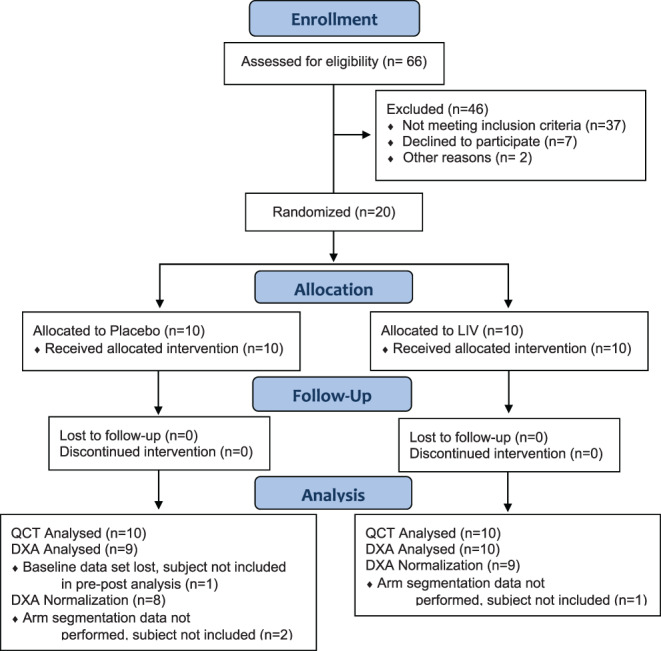
CONSORT diagram for the Duchenne low intensity vibration trial. CONSORT = Consolidated Standards of Reporting Trials.

Sixty‐six patients were screened for the study, with 37 not meeting inclusion criteria and nine who declined to participate (Fig. 1). Across the 20 enrolled subjects (Table 1), diagnosis of DMD was made at 2.8 ± 1.2 years, with steroid therapy begun at 5.1 ± 1.1 years. There were significant differences in age between placebo (6.6 ± 1.8 years) and active (LIV) (9.4 ± 3.4 years) subjects (*p* < 0.05). There were also significant differences in height between placebo (114.0 ± 12.5 cm) and LIV (127.4 ± 15.7 cm) subjects (*p* < 0.05), and a trend in difference in weight between placebo (20.8 ± 4.6 kg) and LIV (31.5 ± 10.2 kg) subjects (*p* < 0.20). Height *Z*‐score for placebo (−1.11 ± 1.0) was not different than LIV (−1.3 ± 1.4). Although assignment of platform status was blinded, these disparities in age, weight, and height remain a major limitation of the study and are addressed in the discussion.

The compliance was evaluated using the patients' diaries and the platforms' records was 79% in LIV and 75% in placebo groups. Specific compliance measures per subject, including logbooks, were lost to follow‐up, and thus efficacy as a function of compliance could not be determined.

### 14‐Month changes in body habitus

When considering subject specific changes in height from baseline, there was a 5.6% increase in the placebo group (*p* < 0.05), as compared to a 4.5% increase in height in the LIV group (*p* < 0.05), but no difference in growth rates between the groups (*p* = 0.27). When considering subject specific changes in weight from baseline, there was a 13.1% increase in the placebo group (*p* < 0.05) as compared to a 17.8% increase in the LIV group (*p* < 0.05), representing a 5.8% difference in body mass gained between the groups (*p* < 0.20).

### Calcium and vitamin D intake

The dietary calcium intake was 650 ± 132 mg/d at baseline and 710 ± 129 mg/d at month 3, as established by phone interview, measures in line with the average calcium intake of healthy Italian children.^(^ ^35^ ^)^ After dietary adjustment at month 3, it increased to 1186 ± 233 mg/d by end of study (*p* < 0.05 versus baseline). The adherence to diet and vitamin D intake was estimated via interviews with children and parents about calcium intake from foods, by measuring the children's' serum 25‐D levels, and by checking the used calcifediol bottles that the patients were asked to keep and bring back. “High adherence,” defined as taking at least 80% of the prescribed doses, was estimated in 73 ± 6.3% of patients, and even those with lower adherence had an increased calcium intake with respect to baseline (average increase: 280 ± 110 mg/d). High adherence to calcifediol treatment was estimated in 84 ± 5.3% of patients. There was no difference between the LIV and placebo groups in any dietary measures (data not shown).

### 14‐Month changes in BSAP, PTH, and OC and vitamin D

Baseline and 14‐month markers of bone turnover are summarized in Table 2. Follow‐up serum measures were lost to follow‐up for five subjects in each group, so that baseline/end‐of‐study comparisons are based on *n* = 5 in each group. Baseline BSAP in the placebo group did not change at 14 months (+1.8%; *p* = 0.27), while the LIV group rose 7.2% (*p* < 0.20). Baseline PTH in the placebo group did not change at 14 months (*p* = 0.47), while rising 12% in the LIV group (*p* < 0.035). Baseline OC did not change at 14‐months in either the placebo group (−2.1%; *p* = 0.40) or LIV group (+16.9%; *p* = 0.33). Baseline 1,25‐D did not change at 14‐months in either placebo (−10.5%; *p* = 0.24) or LIV (+ 6.6%; *p* = 0.32) groups. Baseline 25‐D increased 31% in the placebo group (*p* < 0.20), but did not change in the LIV group (−5%; *p* = 0.42).

**Table 2:** Bone Turnover Markers

	Placebo		LIV	
Parameter	Baseline	14 months	Baseline	14 months
BSAP	51.6 ± 17.9	50.7 ± 23.3	61.2 ± 35.2	65.6 ± 22.3
	−1.8%; *p* = 0.27		+7.2%; *p* < 0.20	
PTH	23.8 ± 7.5	24.1 ± 1.0	29.2 ± 8.2	32.7 ± 10.4
	<1%; *p* = 0.45		+12%; *p* < 0.05	
OC	61.7 ± 9.6	60.4 ± 18.9	77.3 ± 32.0	90.4 ± 37.9
	−2.1%; *p* = 0.40		+16.9%; *p* = 0.33	
1,25‐D	37.7 ± 11.6	33.8 ± 11.5	51.5 ± 11.0	54.9 ± 12.4
	−10.5%; *p* = 0.24		+6.6%; *p* = 0.32	
25‐D	17.5 ± 7.8	23.0 ± 9.8	38.0 ± 17.6	36.0 ± 10.0
	+31.4; *p* < 0.20		−5.3; *p* = 0.42	

### QCT measures of bone density in the tibia

**Fig. 2:**
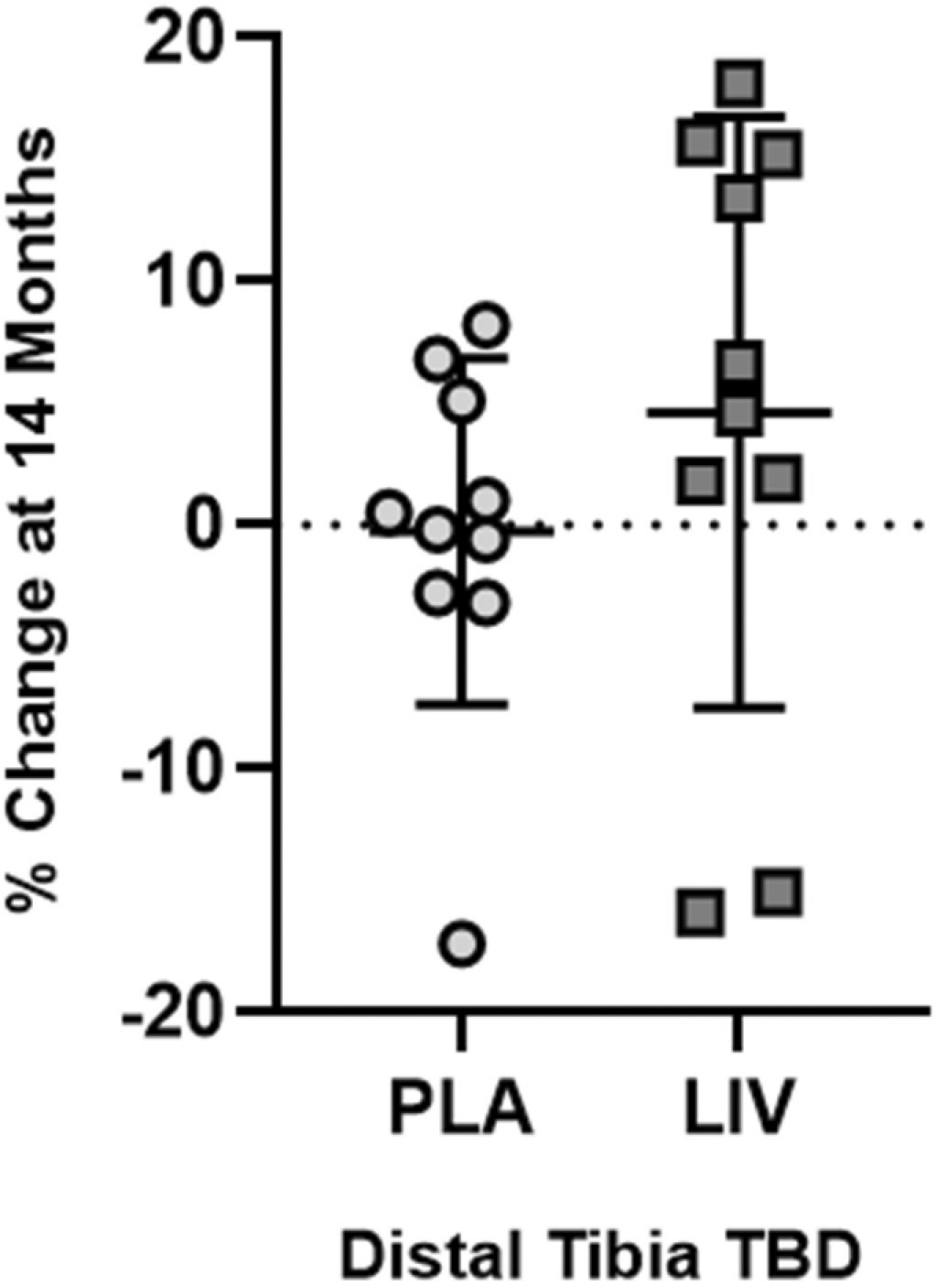
QCT measured changes (mean ± SD) at 14 months in TBD in distal tibia. TBD did not change in placebo (circles, 0.2% below baseline, *p* = 0.43), in some contrast with TBD increases typical to healthy boys of the same age.^(^ ^73^ ^)^ LIV increased TBD by 4.6% (squares, *p* < 0.20 from baseline). Unfortunately, because of the loss of subject‐specific compliance records, the ability to correlate non‐responders (eg, two LIV subjects that lost TBD) to their daily use of the device was not possible.

Data are reported as *n* = 10 in each group. After 14 months, trabecular bone density (TBD) in the proximal tibia remained unchanged in the placebo group (+1.0%; *p* = 0.44), while a 3.5% rise in the LIV group trended toward significance (*p* < 0.20). TBD in the distal tibia remained unchanged in the placebo group (−0.2%; *p* = 0.43), while rising 4.6% in the LIV group (*p* < 0.20, Fig. 2). When considering subject‐specific changes from baseline, TBD of the distal region was 4.9% higher in LIV as compared to the placebo group (*p* < 0.20). Increases in cortical BMD of the tibial midshaft were significant in the placebo group (+1.9%, *p* < 0.20) but failed to reach significance in the LIV group (+0.4%, *p* = 0.55). There was no difference in changes in cortical bone in the placebo as compared to LIV group (*p* = 0.47).

### BMD and BMC measures of TBLH

Data reported are *n* = 9 for both LIV and placebo groups; baseline data sets could not be located for one subject in each group. As measured by DXA, BMD changes in TBLH in the placebo group showed a 3.5% increase over 14 months (*p* < 0.05), as compared to a 2.9% increase in the LIV group (*p* < 0.05). Changes in the two groups were not different from each other (*p* = 0.44). BMC changes in TBLH in the placebo group showed a 15.5% increase over 14 months (*p* < 0.05), as compared to a 15.0% increase in the LIV group (*p* < 0.05). Changes in the two groups were not different from each other (*p* = 0.39).

**Fig. 3:**
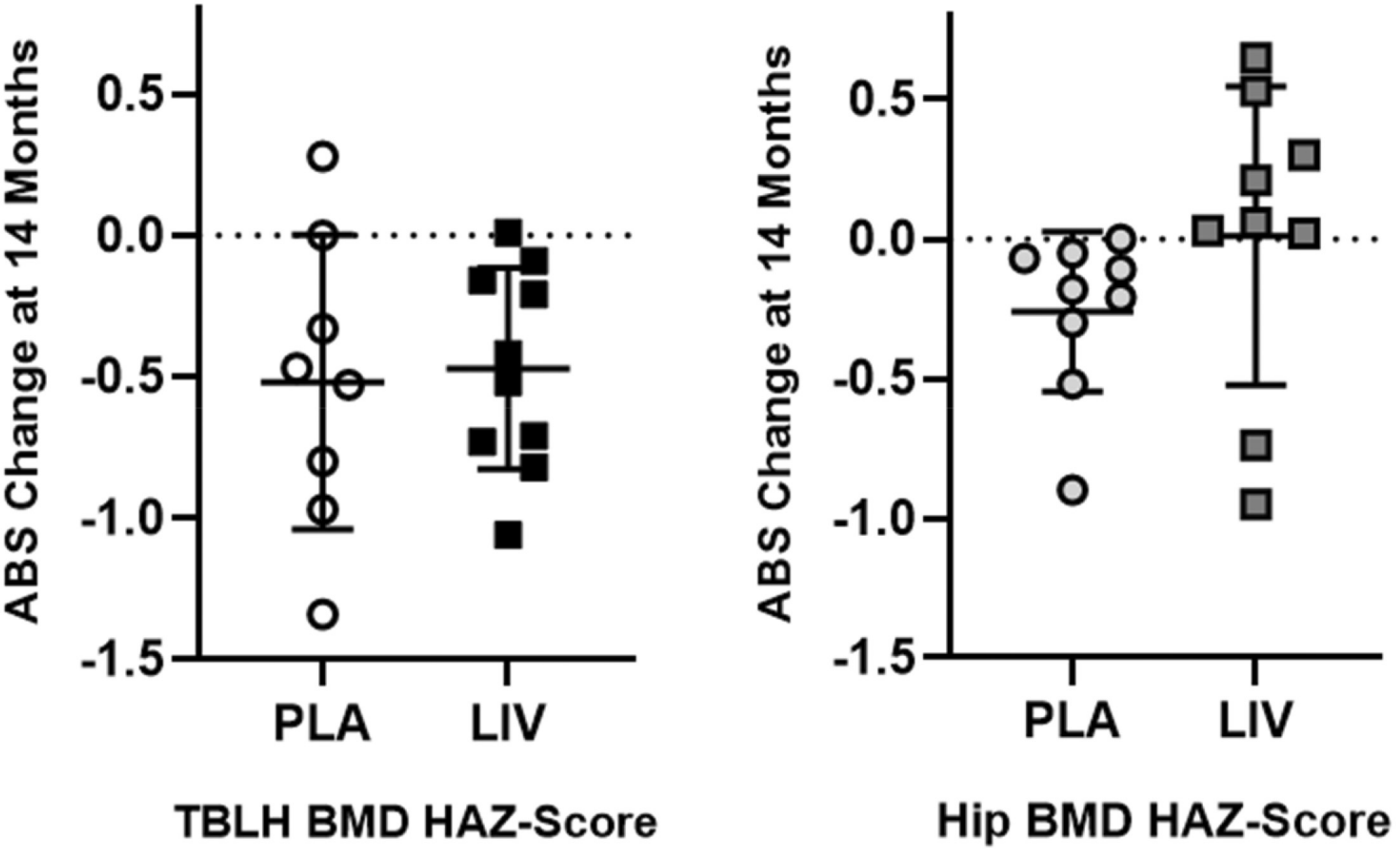
Absolute change in TBLH (left) and hip (right) BMD HAZ score from baseline (mean ± SD). The 14‐month decrease from baseline in TBLH was significant for both placebo and LIV groups (*p* < 0.05), but the differences between groups was not different (*p* = 0.95). In contrast, the absolute decrease in HAZ hip BMD was significant in the placebo group (*p* < 0.05), but there was no change from baseline in LIV.

The degree to which the skeleton of these DMD subjects is compromised becomes evident with direct comparisons to age‐ and height‐matched healthy boys. HAZ of TBLH BMD in the placebo group was −0.3 at baseline and fell to −0.8 by the end of 14 months (*p* < 0.05). HAZ of TBLH BMD of the LIV group at baseline was −1.2 and fell to −1.7 by the end of the experimental period (*p* < 0.05). Changes between groups were not different from each other (*p* = 0.95, Fig. 3). Absolute decreases in HAZ of TBLH BMC were −0.09 in both placebo and LIV, with no differences from baseline or between groups.

### BMD and BMC measures of hip

DXA measures of BMD in the hip of the placebo group did not change over the experimental period (+0.3%; *p* = 0.35), while increasing 2.8% in the LIV group (*p* < 0.20). There was no difference between groups (*p* = 0.24). DXA measures of BMC in the hip increased 8.6% in the placebo subjects (*p* < 0.05) and 12.4% in LIV subjects (*p* < 0.05). There was no difference between groups (*p* = 0.29).

### BMD and BMC HAZ scores of hip

Matched to healthy boys, hip BMD HAZ scores of the placebo group at baseline were −1.4, falling 21% to −1.7 by the end of the 14‐month protocol (*p* < 0.05). Baseline HAZ scores of hip BMD in the LIV group were −2.6, with a 0.9% drop at 14 months, not significantly different from baseline (*p* = 0.91, Fig. 3). HAZ of hip BMC in the placebo group fell 13% (*p* < 0.20) from −2.0 to −2.3, while baseline measure of −3.2 in the LIV group did not change at 14 months (*p* = 0.87). Absolute HAZ in hip BMD fell 0.3 in the placebo group (*p* < 0.05) but remained similar to baseline in the LIV group (−0.02; *p* = 0.45, Fig. 3). Absolute HAZ in hip BMC fell 0.28 in the placebo group (*p* < 0.20) but remained similar to baseline in the LIV group (−0.04; *p* = 0.87).

### BMD and BMC measures of spine

DXA measures of BMD in the spine of the placebo group increased 5.9% (*p* < 0.05), while increasing 7.8% for LIV subjects (*p* < 0.05). Changes in the two groups were not significantly different from each other (+1.5%; *p* = 0.34). DXA measures of BMC in the spine of the placebo group increased 4.7% (*p* ≤ 0.20), whereas BMC in the LIV group increased 8.8% (*p* < 0.05). Changes in the LIV group were not different than the placebo group (+4.3%; *p* = 0.29).

### BMD and BMC HAZ scores of spine

Baseline HAZ of BMD spine in the placebo group was −0.11 at baseline, and did not change by the end of the 14‐month protocol (+0.38; *p* = 0.29). At baseline, HAZ score of spine BMD in LIV was −0.8, and had not changed at 14 months (−0.2, *p* = 0.54). HAZ of spine BMC in the placebo group at baseline was −1.5, and had not changed at 14 months (−14%, *p* = 0.29). HAZ of spine BMC in the LIV group began at −2.1, with no change at 14 months (*p* = 0.89).

### Bone, lean, and fat measures of arm

BMD changes in the left arm of the placebo group showed a 5.6% increase over 14 months (*p* < 0.20), as compared to a 3.1% increase in the LIV group (*p* = 0.20). Changes in BMD in the left arm measured in the placebo group was not different from the LIV group (*p* = 0.49). BMC changes in the left arm in the placebo group showed a 13.4% increase over the 14 months (*p* < 0.05), as compared to a 7.7% increase in the LIV group (*p* < 0.05). Over the 14‐month protocol, BMC in the left arm of the placebo group increased 5.7% more than the LIV group (*p* = 0.20).

Fat mass in the arms of the placebo group increased 15.7% across the 14 months (*p* < 0.05), as compared to 16.6% in the LIV group (*p* < 0.05). Group‐specific changes were not different from each other (*p* = 0.57). Lean mass in the placebo group increased 11.0% across the 14‐month period (*p* < 0.05), in contrast to 5.8% increase in the LIV group (*p* < 0.05). Group‐specific changes were not different from each other (*p* = 0.55).

### Fat and lean mass of TBLH and leg

**Fig. 4:**
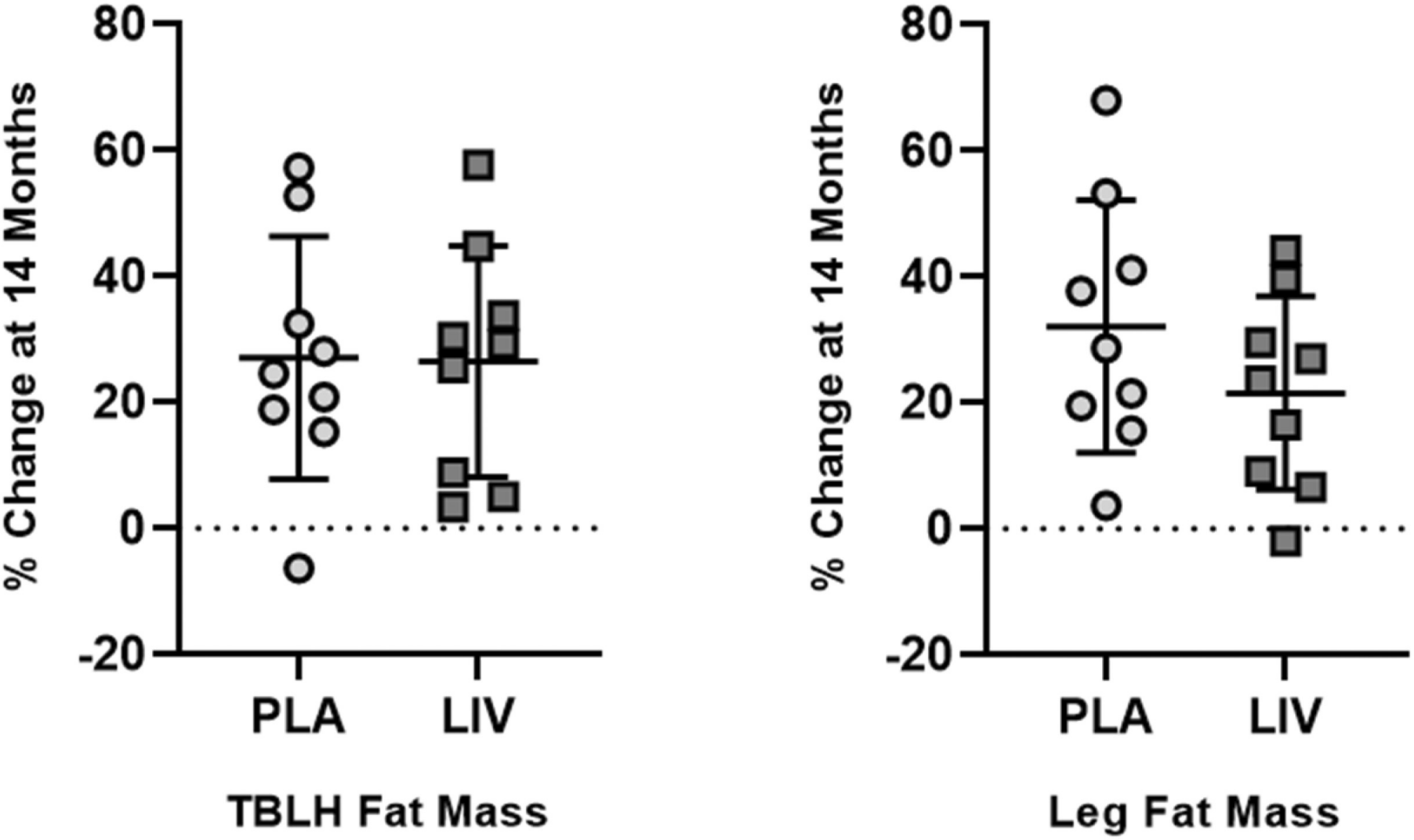
Percent increase (mean ± SD) from baseline in fat mass for TBLH (left) and hip (right). The 14‐month increase from baseline in TBLH was significant (*p* < 0.05) for both placebo (circles) and LIV (squares) groups, but the differences between groups was not different (*p* = 0.95). DXA measured fat mass of left leg showed increases in both placebo (circles; 32.1%) and LIV (squares; 21.6%) DMD subjects. Subject‐specific changes showed a 40% suppression of fat mass in LIV as compared to placebo groups (*p* < 0.20).

DXA‐measured increase in TBLH fat mass across the 14 months were not different (*p* = 0.94) between the placebo group (+27.1%, *p* < 0.05 from baseline) and the LIV group (+26.5%, *p* < 0.05 from baseline). Fat mass of the left leg showed a 32.1% (*p* < 0.05) increase in placebo subjects as compared to a 21.6% (*p* < 0.05) increase in LIV subjects, a 40% suppression of fat accumulation in group‐specific changes (*p* < 0.20; Fig. 4).

DXA‐measured increase in TBLH lean mass across the 14 months was similar (*p* = 0.69) between the placebo group (+11.3%, *p* < 0.05 from baseline) and the LIV group (+9.7%, *p* < 0.05 from baseline). Lean mass of the left leg closely tracked TBLH, with a 10.7% (*p* < 0.05) increase in the placebo group as compared to an 9.5% (*p* < 0.05) increase in the LIV group (*p* = 0.75 between groups).

### Spine and hip BMD and BMC normalized to TBLH

Post hoc analyses of DXA data were first performed by normalizing subject‐specific parameters to that individual's changes measured across the entire body minus the head (TBLH), helping determine if LIV influenced those regions of the skeleton that were subject to the mechanical signal delivered primarily to the weight‐bearing bones.^(^ ^28^ ^)^ Normalized to changes in TBLH, BMD in the spine of the placebo group was not different to baseline (+2.5%; *p* = 0.42), as compared to a 4.1% increase in the LIV group (*p* < 0.20). There were no differences between groups (*p* = 0.34). When normalized to changes in TBLH, BMC in the spine dropped 6.0% (*p* < 0.05) in the placebo group, as contrasted to a 0.4% decrease in the LIV group (*p* = 0.39). When considering subject‐specific changes of BMC of spine from baseline, there was a 5.6% positive swing from the placebo group to LIV (*p* < 0.20).

Relative to TBLH, BMD in the hip fell 3.0% (*p* < 0.05) in the placebo group, while remaining unchanged from baseline in the LIV group (−0.9%; *p* = 0.40). Group‐specific changes were not different from each other (*p* = 0.43). Normalizing BMC changes in TBLH to those measured in the hip showed a 5.4% drop in BMC in the placebo group (*p* < 0.20) as compared to no change from baseline in the LIV group (−0.8%; *p* = 0.78). Group‐specific changes were not different from each other (*p* = 0.24).

### Spine and hip BMD and BMC normalized to arm

**Fig. 5:**
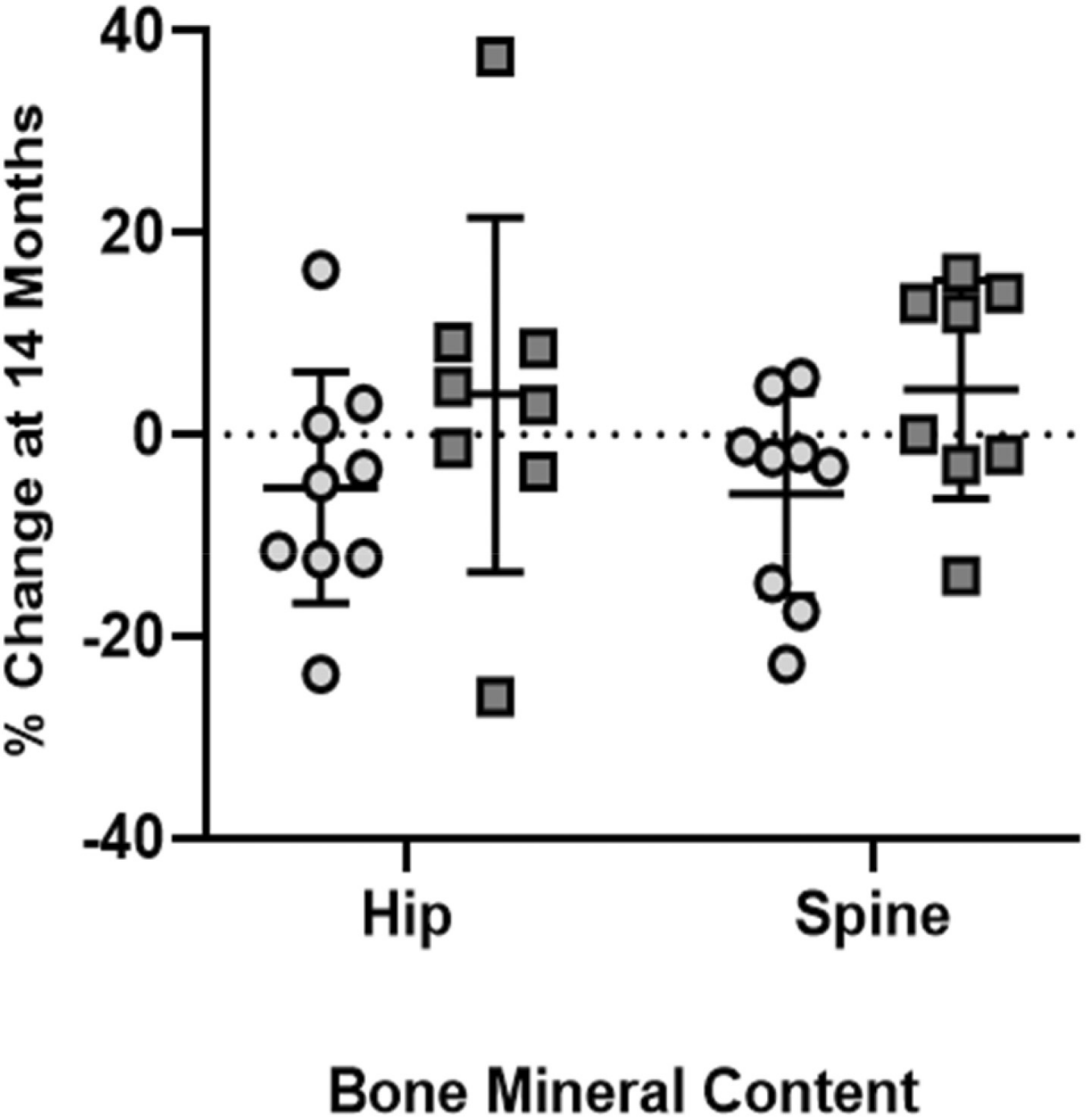
DXA measured percent change (mean ± SD) in BMC at hip and spine at 14 months within placebo (circles) and LIV (squares) subjects, as normalized to changes in arm (intrasubject control). Hip BMC in placebo decreased by 5.2%, while the LIV group increased by 4.0%, a 9.2% shift (*p* < 0.20). BMC in spine dropped 5.9% in placebo, and increased 4.4% in LIV, a 10.2% shift (*p* < 0.05).

**Fig. 6:**
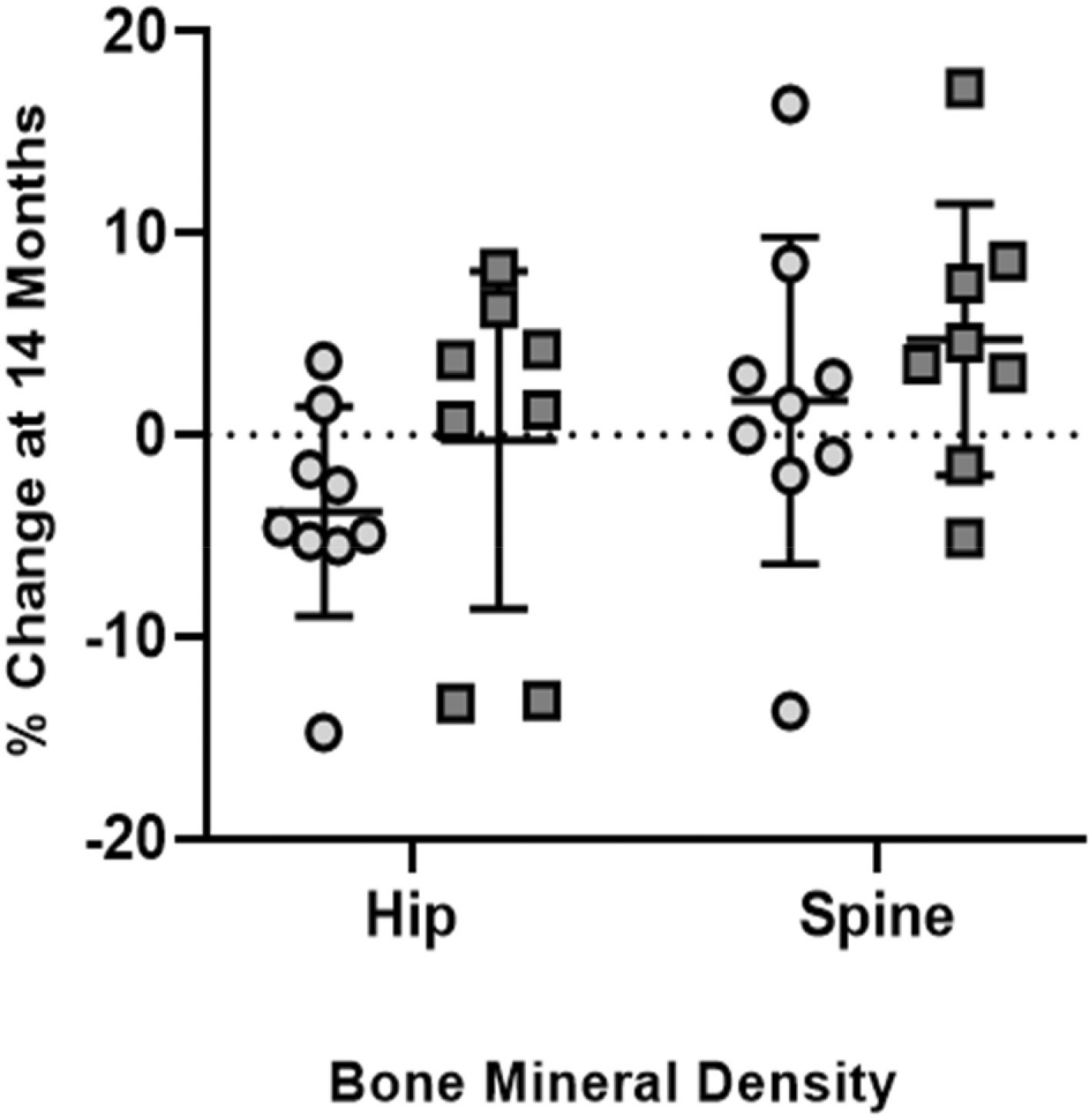
DXA measured percent change (mean ± SD) at 14 months in BMD in placebo (circles) and LIV subjects (squares), as normalized to changes in arm (intrasubject control). Hip BMD in the placebo decreased by 3.8%, while LIV remained unchanged (0.02%), a 3.5% shift (*p* < 0.20). BMD spine increased 1.7% in placebo, and 4.7% in LIV, a 3.0% shift (*p* < 0.20).

Post hoc analyses of DXA data were also performed where subject specific parameters were normalized to that subject's changes measured in the arm. When normalized to changes in the arm, BMD in the spine of the placebo group was not different from baseline (+1.7%; *p* = 0.52), as compared to a 4.7% (*p* < 0.20) increase in the LIV group (Fig. 5). Group‐specific changes were not different from each other (*p* = 0.21). When normalized to changes in the arm, BMC in the spine dropped 5.9% (*p* < 0.20) in the placebo group, as contrasted to a 4.4% increase in the LIV group (*p* < 0.20), a 10.2% positive swing from the placebo to the LIV groups (*p* < 0.05; Fig. 6).

Normalizing BMD changes relative to those realized in the arm across 14 months to those measured in the hip showed a 3.8% fall in the placebo group (*p* < 0.05), while remaining unchanged from baseline in the LIV group (−0.2%; *p* = 0.80), representing a 3.5% difference between the groups (*p* < 0.20; Fig. 5). BMC in the hip normalized to that measured in the arm, there was a 5.2% drop in the placebo group (*p* < 0.20), in contrast to no change from baseline in the LIV group (+4.0%, *p* = 0.26), a 9.7% positive swing from the placebo to the LIV groups (*p* < 0.20; Fig. 6).

#### Fractures

At baseline, across the whole group, 13 lower‐limb fractures had been sustained by eight DMD subjects before starting the study (40%). No vertebral fractures were reported in the clinical records, but baseline lateral spine radiographs revealed six prior fractures of dorsal vertebrae in four DMD subjects (one fracture each in three patients; three fractures in one patient).

**Fig. 7:**
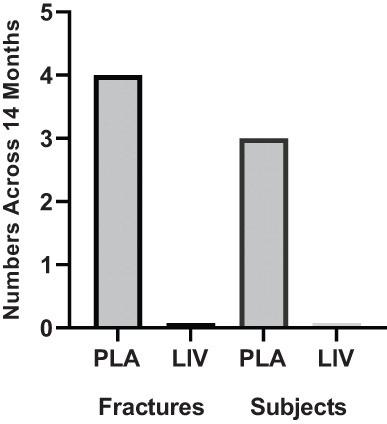
Absolute number of incident fractures (left) and subjects with fracture (right) identified across the 14‐month protocol in both placebo and LIV group (*N* = 10 subjects in each group). Although the causes of the fractures are not known, the difference between groups is significant (*p* < 0.05).

Regarding incident fractures at the end of the protocol, there was one dorsal vertebral fracture and three appendicular fractures (two foot fractures, one fibula fracture) identified in three patients in the placebo group (30%), while no new fractures were identified in LIV, a significant difference between groups (*p* < 0.05; Fig. 7). The cause of these fractures is not known.

## Discussion

Implementation of exercise programs to protect the musculoskeletal systems of children with DMD has been complicated by fears that high‐intensity or eccentric exercise may accelerate muscle deterioration, that active weight‐bearing protocols are often beyond the sustainable reach of this population, or—paradoxically—that these activities may increase risk of fractures.^(^ ^41^ ^)^ As a surrogate for exercise protocols, this prospective randomized double‐blind, placebo‐controlled study reports on the effects of LIV on bone status in children with DMD. The 20 DMD boys enrolled in this study were all ambulant, treated with the same GC regimen, with dietary calcium intake according to the RDA for age, and receiving calcifediol (25‐OH vitamin D_3_) supplementation. This study showed that LIV was safe and well tolerated, because daily compliance was high and there were no reported adverse events. The study, although small, provides some insight into the potential of exogenously delivered, low‐magnitude mechanical signals as a means of protecting the skeleton in an at‐risk population.

LIV has been shown to increase bone mass and quality in children with disabling conditions, including cerebral palsy^(^ ^42^ ^)^ and adolescent girls with idiopathic scoliosis.^(^ ^43^ ^)^ LIV augments bone accretion in survivors of childhood cancer^(^ ^44^ ^)^ and patients with Crohn's disease.^(^ ^45^ ^)^ In a 1‐year study on young women (age 15–21 years) with osteoporosis, LIV was shown to be anabolic to both femur and spine, as well as paraspinous musculature, representing improvements relative to that measured in placebo control and achieved while suppressing fat formation in the torso.^(^ ^27^, ^46^ ^)^ Translating LIV to children with muscular dystrophies, a 1‐year pilot trial evaluated LIV as a protective influence on muscle function in five patients with DMD or Becker muscular dystrophy, with the first 6 months exposing each subject to 10 minute/d of LIV (0.4 g at 30 Hz), with the second 6 months halting the LIV intervention.^(^ ^29^ ^)^ Timed motor function and lower extremity muscle strength remained unchanged or slightly improved during the intervention phase, but was followed by marked deterioration once LIV was discontinued. Although the investigators concluded LIV to have a “stabilizing effect on lower extremity muscle function,” no measures on the skeletal system were performed.

In the DMD study reported here, changes from baseline to 14‐month DXA measures of BMC and BMD in TBLH, arm, hip, and spine showed significant increases in both the LIV and placebo groups, emphasizing that the skeleton of these children continued to grow. Despite differences in ages of the two groups, 14‐month increases in BMD and BMC measures of TBLH and arm were highly similar between LIV and placebo subjects, suggesting that skeletal growth in the non‐weight‐bearing regions progressed in a similar fashion. Nevertheless, even with increases in BMD and BMC in LIV and placebo subjects, comparative metrics of DMD bone quality and quantity—as established by HAZ—fall well short of that observed in healthy children, indicating that the DMD skeleton is more susceptible to fracture,^(^ ^47^ ^)^ a risk that becomes more severe with age and extended GC use.^(^ ^48^ ^)^

HAZ scores provide information about the skeleton relative to an age‐ and height‐adjusted average established in healthy boys.^(^ ^37^ ^)^ For example, the Hip BMD HAZ for this DMD cohort shows the baseline measures for LIV subjects being at −2.45, more than two SDs below what might be expected at that age and for that height. And while HAZ declined 21% in the placebo group, pointing toward an escalating susceptibility to fracture, the LIV group remained unchanged from baseline, pointing to a potential for LIV to limit bone loss in the weight‐bearing skeleton of high‐risk DMD subjects. These regional *Z*‐scores reinforce prior findings that skeletal quality in the lower appendicular skeleton is below that of the spine or total body,^(^ ^49^ ^)^ but are encouraging in that LIV suppresses further decline in these regions of greatest risk.

**Fig. 8:**
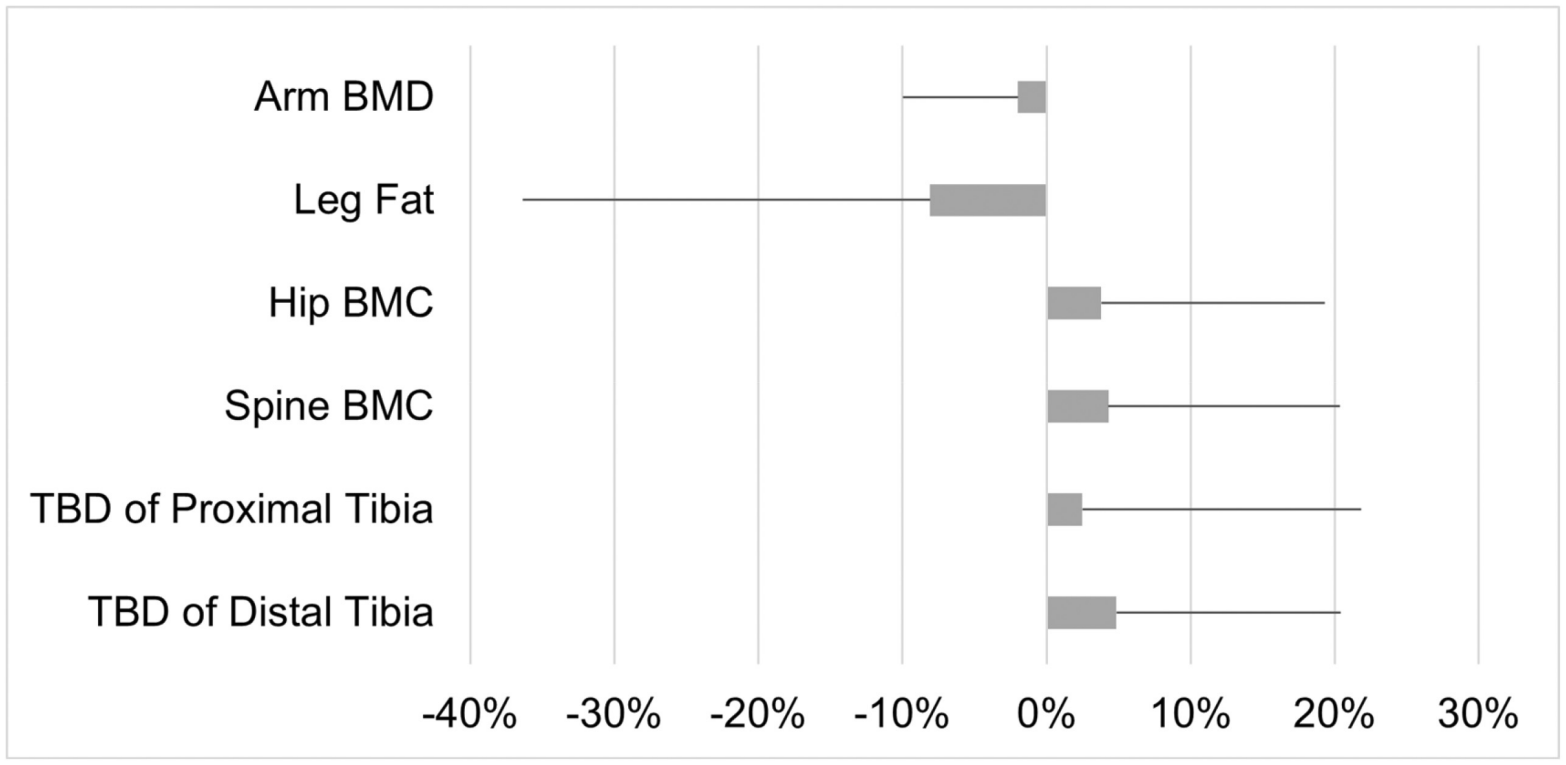
Mean difference (%) in 14‐month changes between LIV and placebo subjects. The error bars represent the standard error of the difference, calculated as the square root of the sum of squared SDs of the 14‐month changes in LIV and control subjects. Although fat mass in the leg of LIV subjects decreases relative to placebo subjects, parameters directly related to bone quantity/quality increase. The small differences in changes at the arm between LIV and placebo subjects are intended to represent an internal control, emphasizing that this region changes very little across 14 months.

When considering the aggregate influence of LIV on DMD, trends in the skeleton showed relative increases in both BMD and BMC at the hip, spine, and tibia in the LIV versus the placebo group, while gains in fat mass in the lower limb of the LIV group were lower than the placebo group (Fig. 8). When these measures were normalized, first to the overall index of TBLH, and then to 14‐month changes measured in the arm, the consequences of DMD to the weight‐bearing skeleton became more apparent within the placebo group, with neither the hip nor spine “keeping up” with BMC and BMD increases in upper regions of the body. This failure of the weight‐bearing bones to parallel increases in the arm is—at least in some part—a consequence of diminished functional demands made to these regions: as the disease progresses, those individuals with DMD are less active and not loading their skeleton in the same way that healthy boys might do.^(^ ^21^ ^)^ Thus, although genetic programming drives the skeleton to continue to grow across adolescence,^(^ ^37^ ^)^ robustness, the added anabolic benefit of mechanical loading, is less evident, resulting in a mismatch between bone quantity and quality in the upper and lower extremities.^(^ ^14^ ^)^

Normalizing bone quality and quantity in the hip and spine of LIV subjects to either TBLH or the arm suggest that these mechanical signals could serve as a protective agent, particularly when compared to the regional disparities identified in the placebo group. For example, when normalized to specific changes in TBLH occurring over the 14 months, BMC in the spine increased by 1% in the LIV group, but fell 6.2% in the placebo subjects, representing a 5.6% “benefit” of LIV even when considering that this older cohort is at greater risk. Of course, TBLH *includes* the spine and hip, and thus any changes in these regions of interest would be masked by this “aggregate” measure. Normalizing solely to the arm showed that the spine in placebo subjects had decreased 5.6% 14 months later, whereas there was a 4.8% increase in LIV subjects, reflecting a 10.2% benefit of low‐magnitude mechanical signals.

Prior studies using high‐intensity, whole‐body vibration (WBV; >5.0 g) in DMD have not shown responses in bone, perhaps because these studies were short in duration (4, 8, and 12 weeks).^(^ ^50^, ^51^, ^52^ ^)^ In an 8‐week study on 14 DMD patients and eight with spinal muscular atrophy (SMA), high‐intensity WBV for 3 minutes/d, 5×/week, aimed to improve muscle strength and function. Mild functional improvements were observed, including a significant improvement in the 6‐minute walking test in children with SMA,^(^ ^51^ ^)^ but no skeletal measures were made. A 12‐week study examined the influence of high‐intensity WBV delivered 2×/week on muscle and bone in six ambulatory DMD patients,^(^ ^50^ ^)^ but no significant changes in bone mass or strength were measured. Finally, 4 weeks of high‐intensity WBV, delivered 3×/week, in four DMD patients showed the subjects tolerated the intervention, but no significant changes in functional mobility were identified.^(^ ^52^ ^)^ Although the magnitude of the vibration in these studies are up to 20× higher than those used here (eg, 8.0 g versus 0.4 g), it is not clear that a benefit of higher intensity ultimately outweighs the added risk.^(^ ^29^ ^)^ The safety of chronic exposure to high‐intensity vibration must also be considered,^(^ ^53^ ^)^ particularly in those with skeletons at high risk of fracture.^(^ ^54^ ^)^ Together, these data suggest that daily use is a critical ingredient in bone's mechanoresponse, and that efficacy may even be improved by transiently improving the bone cell mechanosensitivity by incorporating multiple bouts per day.^(^ ^55^ ^)^

As measured by DXA, there were significant 14‐month increases in fat mass in TBLH and the arms of both LIV and placebo subjects, with no differences between groups, suggesting LIV had no influence on fat phenotype in non‐weight‐bearing regions of the body. In some contrast, DXA pointed to a 33% increase of fat mass in the leg of placebo subjects, 60% greater than that measured in LIV subjects. Thus, although fat mass increases were significant in the leg of both the LIV and placebo groups, it was also evident that the placebo group rose at a higher rate, a predictor of regional functional decline.^(^ ^56^ ^)^ And while 14‐month increases in lean mass of the leg were also significant in both LIV and placebo subjects, the 2% difference between groups was not different. Importantly, fat encroachment into muscle is a major complication in DMD, starting in the lower limbs as early as the age of 5 years,^(^ ^57^ ^)^ compromising both function and regenerative capacity.^(^ ^58^ ^)^ Although this study was not structured to examine either the fat or muscle phenotypes per se, there is certainly evidence that inactivity is permissive to increased fat production in growing children,^(^ ^59^ ^)^ that GC can promote adipogenesis and suppress osteoblastogenesis in DMD patients,^(^ ^60^, ^61^ ^)^ and that LIV suppressed regional adipogenesis.^(^ ^23^, ^46^ ^)^

As a more precise assessment of bone quality, QCT measures of trabecular bone density of the proximal and distal tibia showed that the placebo group remained essentially unchanged over the course of 14 months, suggesting the structural elements of the bone are not keeping pace with growth patterns in these children.^(^ ^62^ ^)^ In contrast, the LIV group subjects show an increase in TBD across the 14‐month period, suggesting that the anabolic potential of LIV is reinforcing trabecular structures in this region, similar to that measured in children with Crohn's disease^(^ ^45^ ^)^ and postmenopausal women subject to LIV.^(^ ^63^ ^)^ Perhaps such improvements translate to a more robust skeleton and an overall decrease in susceptibility to fracture.^(^ ^64^ ^)^

There is a large fracture burden in children with DMD. Results reported from the NorthStar data base show that—over a 4‐year period—incident fractures occurred in 28% of the 564 participants, with this rate almost doubling in those patients taking corticosteroids.^(^ ^48^ ^)^ Although GC use has helped preserve muscle mass and function in DMD,^(^ ^65^ ^)^ consequences of long‐term use include accelerated decline of the skeleton and greater risk of fracture. Indeed, 30% of the subjects in the placebo group suffered an incident fracture over the 14‐month period of the study. In some contrast, despite HAZ scores well below those of the placebo group, there were no incident fractures reported in the active cohort. It is possible that no fractures in the active group is a coincidence, or perhaps the influence of these mechanical signals serves to improve bone microarchitecture, and thus provides a proportional benefit to fracture resistance.^(^ ^63^ ^)^ Further, as Petryk and colleagues^(^ ^29^ ^)^ demonstrated an improvement of muscle function and strength through LIV, perhaps these small contributions work synergistically, through stability, balance, and skeletal strength, to resist fractures. Regardless, any means of reducing fracture incidence can have a tremendous impact on retaining quality‐of‐life, because over 40% of ambulatory DMD patients who suffer a fracture never return to weight‐bearing activities.^(^ ^14^ ^)^

This double‐blind, prospective trial was designed to determine if LIV could suppress deleterious changes in the DMD skeleton. Several limitations must be considered in interpreting the results. First and foremost, the study was designed such that the active/placebo devices were randomly assigned. Unblinding revealed that the mean age of the LIV group was 3 years older than that of the placebo group. Although such disparities are always a risk in a small trial,^(^ ^40^ ^)^ the observation that older DMD subjects comprising the LIV group were building bone and suppressing fat production can also be considered reassuring, as these boys—by any perspective—would be less active, at greater fracture risk, and at a later stage of disease progression. Further, reporting the 14‐month outcomes in relation to HAZ scores, helps diminish age‐specific differences between groups, particularly because the BMD/BMD decreases in TBLH were identical between the LIV and placebo groups. Most important, it is encouraging that the fracture incidence in the LIV group was significantly lower than the placebo group, despite a higher age and lower baseline BMD. And finally, it is disappointing, indeed, that individual compliance records were lost, nullifying any ability to identify a dose:response relationship,^(^ ^27^ ^)^ and determine if nonresponders were perhaps using the device less frequently (Fig. 3).

LIV is only a potential surrogate for exercise, not a replacement.^(^ ^66^ ^)^ LIV's efficacy is stronger in some populations than others,^(^ ^67^ ^)^ with those that are “least” responsive tending toward older cohorts, including the frail elderly.^(^ ^68^ ^)^ That the DMD group is young, however, can only potentiate LIV's ability to combat skeletal decline.^(^ ^27^, ^42^, ^43^, ^44^, ^45^ ^)^ Further studies are necessary to determine if—and how—LIV can protect muscle strength and function in the human, and whether this intervention could work synergistically with drug interventions designed to restore muscle composition.^(^ ^69^ ^)^ Prior work in the mouse has shown that LIV can stimulate hypertrophy in muscle,^(^ ^70^ ^)^ and can promote power output in muscle units,^(^ ^71^ ^)^ whereas satellite cells, compromised by endocrine deficiency, are protected by LIV.^(^ ^72^ ^)^ Nevertheless, until clinical studies are performed, it is impossible to project whether LIV can guard the musculoskeletal “system” against DMD. Results from this 14‐month study suggest that daily intervention with LIV is safe, well tolerated, and may benefit the quantity and quality of the weight‐bearing skeleton of DMD children, and thus reduce their risk of fracture.

## In Memoriam

It is with a great sense of sorrow that we must write that the lead clinical investigator, and first author of this study, Professor Maria Luisa Bianchi, passed away in September 2020. She was an internationally renowned expert on pediatric metabolic bone disease, fully committed to clinical research that could ultimately help her patients and families of those children. We are grateful for her leadership and enthusiasm for fostering this study forward, and hope that, in some small way, this work contributes to her lasting memory and enormous impact on the science and community of pediatric endocrinology.
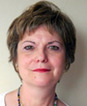


## Author Contributions

**Maria Luisa Bianchi:** Conceptualization; investigation; methodology; project administration; supervision; writing – original draft. **Silvia Vai:** Investigation; methodology; project administration. **Giovanni Baranello:** Investigation; methodology; project administration. **Francesca Broggi:** Investigation; methodology; project administration. **Stefan Judex:** Formal analysis; methodology. **Thomas N. Hangartner:** Methodology; software; supervision; validation; writing – original draft; writing – review and editing. **Clinton T Rubin:** Conceptualization; formal analysis; investigation; methodology; project administration; validation; writing – original draft; writing – review and editing.

## Conflicts of Interest

CTR and SJ have several U.S. and international patents issued on the use of low intensity vibration for the treatment of musculoskeletal injury and disease. CTR is also a founder of Marodyne, Inc., who developed the LIV platform for clinical use as LivMD. No other authors have any conflicts to report.

### Peer review

The peer review history for this article is available at https://publons.com/publon/10.1002/jbm4.10685.

## Data Availability

The data that support the findings of this study are available on request from the corresponding author. The data are not publicly available due to privacy or ethical restrictions.
